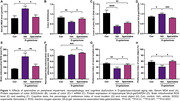# Spermidine Treatment Attenuates Systemic and Gut Oxidative Stress, Neuropathology and Cognitive Deficits in D‐galactose Induced Aging Rats

**DOI:** 10.1002/alz.086335

**Published:** 2025-01-03

**Authors:** Napatsorn Saiyasit, Wasana Pratchayasakul, Napapan Kangwan, Wichida Kaorop, Chayadom Maneechote, Chanon Kunasol, Nattapat Ngowthammatas, Busarin Arunsak, Suriphan Donchada, Aphisek Kongkaew, Nipon Chattipakorn, Siriporn C Chattipakorn

**Affiliations:** ^1^ Neurophysiology unit, Cardiac Electrophysiology Research and Training Center, Faculty of Medicine, Chiang Mai University, Chiang Mai Thailand; ^2^ Center of Excellence in Cardiac Electrophysiology Research, Chiang Mai University, Chiang Mai Thailand; ^3^ Neurophysiology Unit, Cardiac Electrophysiology Research and Training Center, Faculty of Medicine, Chiang Mai University, Chiang Mai Thailand; ^4^ Department of Physiology, Faculty of Medicine, Chiang Mai University, Chiang Mai Thailand; ^5^ Division of Physiology, School of Medical Sciences, University of Phayao, Phayao Thailand; ^6^ Research Administration Section, Faculty of Medicine, Chiang Mai University, Chiang Mai, Chiang Mai Thailand; ^7^ Department of Oral Biology and Diagnostic Sciences, Faculty of Dentistry, Chiang Mai University, Chiang Mai Thailand

## Abstract

**Background:**

Our previous studies reported that D‐galactose (D‐gal) administration for four to eight weeks caused metabolic disturbance, brain mitochondrial dysfunction, and brain aging, leading to cognitive dysfunction in similar with natural aging condition. Spermidine is a polyamine that can be found naturally. Spermidine has been showed the beneficial effects on various models, such as attenuating metabolic/gut impairments in obesity, and ameliorating memory loss in aged model. However, those beneficial effects of spermidine in D‐galactose‐induced aging condition is still unclear.

**Method:**

Eighteen female Wistar rats were divided into two groups which received either NSS as a control group (n = 6) or D‐gal (150 mg/kg/day, n = 12), subcutaneously for total twenty weeks. At week 12, D‐gal‐treated rats were divided into two subgroups; NSS as a vehicle and spermidine (20 mg/kg/day), orally for 8 weeks (n = 6 per subgroup). At the end of protocol, the cognitive tests; novel object location (NOL) and novel object recognition (NOR) were performed. After animals were euthanized, brain/gut tissues and blood samplings were collected to determine the biochemical parameters.

**Result:**

Systemic/gut oxidative stress by increasing the serum malondialdehyde (MDA) level and attenuating colon SOD2 protein expression respectively were shown in D‐gal‐treated rats (**Figure 1A‐B**). Levels of colon ZO‐1 mRNA was decreased in D‐gal‐treated rats, in comparison to control group (**Figure 1C**). Upregulation of hippocampal senescence‐associated b‐galactosidase (SA‐b‐gal), elevation of brain mitochondrial ROS level, and cognitive impairment by reducing the percentage of preference index of NOL and NOR were also observed in D‐gal‐treated rats (**Figure 1D‐E, G‐H**). Interestingly, spermidine treatment in D‐gal‐treated rats alleviated serum MDA and upregulated colon SOD2 protein levels, compared to D‐gal‐treated rats with vehicle (**Figure 1A‐B**). Spermidine also reduced hippocampal SA‐b‐gal protein expression, decreased brain mitochondrial ROS level and swelling (**Figure 1D‐F**), and attenuated the learning and memory impairment by restoring the percentage of preference index of NOL and NOR in D‐gal‐treated rats (**Figure 1G‐H**).

**Conclusion:**

Our findings suggest that spermidine exerts the beneficial effects on cognition through reducing systemic/brain oxidative stress in D‐galactose‐induced aging rats. Therefore, it is possible that spermidine should be considered as a novel therapeutic option for ameliorating neuropathology in aging condition.